# Mental Health of Unaccompanied Immigrant Youth

**DOI:** 10.1192/j.eurpsy.2022.150

**Published:** 2022-09-01

**Authors:** N. Morales, F. Collazos, A. Qureshi

**Affiliations:** Hospital Universitari Vall d’Hebron, Department For Psychiatry, Barcelona, Spain

**Keywords:** Unaccompanied, Migrant, Mental, health

## Abstract

The number of UMY in Spain is increasing, since the early 1990s, mostly coming from the Maghreb, although the number of those coming from different sub-Saharan African countries has gradually increased. Most of them leave their countries fleeing poverty, violence, and in search of better opportunities. They may be influenced by traumatic experiences and social stressors that can lead to emotional distress and mental health problems. They have particular needs and characteristics, so the local Child Protection Systems need to adapt their procedures to facilitate the youngsters’ social Integration and psychosocial development. This presentation will describe an ongoing project being carried out in Catalonia, the main objective is to guarantee the right to mental health of UMY in the Protection System through culturally competent biopsychosocial care, and to effectively coordinate care between the public mental health network and the Child Protection System. Finally, through training and the acquisition of competencies, the aim is to avoid burnout in professionals who care for these youths on the front line. The approach is consistent with the cultural consultation models developed in Montreal and London with the goal of providing structural support for localized and culturally competent responses. This project, to be developed over two years, has four main subprojects: 1. On-line training for professionals in “Cultural competence in mental health and psychosocial intervention”. 2. Training of “peer” UMY as “Community Mental Health Agents”. 3. Creation and implementation of multidisciplinary groups of psychosocial intervention. 4. Culturally competent psychiatric and psychological assessment.

**Disclosure:**

No significant relationships.

